# Influence of socioeconomic status on community-acquired pneumonia outcomes in elderly patients requiring hospitalization: a multicenter observational study

**DOI:** 10.1186/1471-2458-10-421

**Published:** 2010-07-15

**Authors:** Conchita Izquierdo, Manuel Oviedo, Laura Ruiz, Xavier Sintes, Isabel Vera, Manel Nebot, Jose-María Bayas, Jordi Carratalà, Wenceslao Varona, Dolores Sousa, Jose-Miguel Celorrio, Luis Salleras, Angela Domínguez

**Affiliations:** 1 Department of Health, Generalitat of Catalonia, Roc Boronat 81-95, 08005 Barcelona, Spain; 2 Department of Statistics and Operative Research, University of Santiago de Compostela, 15782 Santiago de Compostela, Spain; 3 CIBER Epidemiología y Salud Pública (CIBERESP) Dr Aiguader 88, 08003 Barcelona, Spain; 4 Public Health Agency of Barcelona, Pl Lesseps 1, 08023 Barcelona, Spain; 5 Hospital Clínic, Department of Preventive Medicine and Epidemiology, Calle Villarroel 170, 08036 Barcelona, Spain; 6 Bellvitge University Hospital-IDIBELL, Department of Infectious Diseases, University of Barcelona, C/Feixa Llarga S/N 08907 L'Hospitalet de LLobregat, Barcelona, Spain; 7 Hospital Royo Villanova, Department of Preventive Medicine, San Gregorio 30, 50015 Zaragoza, Spain; 8 Hospital Juan Canalejo, Department of Infectious Diseases, As Xubias, número 84, 15006 A Coruña, Spain; 9 Hospital Ernest Lluch, Department of Preventive Medicine, ctra. Sagunto-Burgos Km 254, 50300 Calatayud, Spain; 10 Department of Public Health, University of Barcelona, C/Casanova 143, 08036 Barcelona, Spain

## Abstract

**Background:**

The associations between socioeconomic status and community-acquired pneumonia outcomes in adults have been studied although studies did not always document a relationship.

The aim of this multicenter observational study was to determine the association between socioeconomic status and community-acquired pneumonia outcomes in the elderly, in the context of a public health system providing universal free care to the whole population.

**Methods:**

A total of 651 patients aged ≥65 years hospitalized due to community-acquired pneumonia through the emergency departments of five Spanish public hospitals were recruited and followed up between May 2005 and January 2007. The primary outcomes studied were: length of stay, intensive care unit admission, overall mortality and readmission. Socioeconomic status was measured using both individual and community data: occupation [categorized in six social groups (I, II, III, IVa, IVb and V)], educational level (≤ primary level or ≥ secondary level) and disposable family income of the municipality or district of residence [>12,500 € (high municipality family income) and ≤12,500 € (low municipality family income)]. The six social groups were further categorized as upper/middle social class (groups I-IVb) and lower class (group V).

Bivariate and multivariate analyses were performed. OR and their 95% confidence intervals were calculated. All statistical tests were two tailed and statistical significance was established as p < 0.05.

**Results:**

17.7% of patients lived in a municipality or district with a high municipality family income and 63.6% were upper/middle social class (I-IVb). Only 15.7% of patients had a secondary education. The adjusted analysis showed no association between pneumonia outcomes and social class, educational level or municipality family income. However, length of stay increased significantly in patients in whom the factors, living alone and being a smoker or ex-smoker coincided (p < 0.001).

**Conclusions:**

We measured socioeconomic status using both individual and community data and found no association between social class, educational level or municipality family income and the variables of pneumonia outcomes. The lack of differences between social classes supports the provision of universal, equitable health care by the public health system.

## Background

Community-acquired pneumonia (CAP) is an important cause of morbidity and mortality in elderly people and those of any age with underlying diseases [1]. In Spain, the overall incidence in adults ranges between 2 and 10 cases/1,000 persons/year in all ages and between 3.16 and 35/1,000 persons/year in persons aged ≥70 years [2-4]. In a Spanish study, the incidence rates increased significantly in the elderly according to age (9.9/1,000 in people aged 65-74 years versus 29.4 in people aged ≥ 85 years) [4].

Hospitalizations due to CAP increase with age and may reach 67% to 75.1% in people aged > 65 years [4,5]. The case-fatality rate of CAP requiring hospital admission in people aged ≥65 is around 12% [6,7] and may reach 17% in people aged ≥ 75 years [5], with higher rates in people with underlying diseases [1,5]. For this reason, the influence of factors related to the prognosis of the disease has been investigated [8,9].

The associations between socioeconomic status and CAP outcomes in adults have been studied although the findings were inconsistent [10-13]. A French study found low socioeconomic status was an independent predictor of significantly longer hospitalization [10], but other reports found no relationship [11]. Jasti *et al*. and Mc Gregor *et al. *found a relationship between hospital readmission and a poor socioeconomic status [11,13] but neither was able to conclude that low socioeconomic status increased CAP mortality [10,12].

In Spain, the associations between socioeconomic status and the use of health services have been studied [14,15] but there is no study on pneumonia outcomes. The main aim of this multicenter observational study was to determine the possible association between socioeconomic status and CAP outcomes in the elderly requiring hospitalization in the context of a public health system providing universal free care to the whole population. Outcomes studied were intensive care unit (ICU) admission, length of hospital stay (LOS), mortality in the first 30 days after admission, and readmission in the first 30 days after discharge.

## Methods

### Study design and setting

A multicenter study was conducted in patients aged ≥65 years recruited in the context of an observational study to assess the effectiveness of the 23-valent polysaccharide vaccine [16]. Patients hospitalized with CAP through the emergency departments of five public hospitals (providing universal free care) in three Spanish regions (Aragon, Catalonia and Galicia) between May 2005 and January 2007 were prospectively recruited and followed up. Briefly, in Spain each person is assigned a reference hospital by geographical area. This includes emergency treatment, referrals from primary health care, acute admissions and programmed surgery. All five hospitals are teaching hospitals. Three are large general hospitals serving an almost-entirely urban population with between 861 and 1400 beds and between 35,747 and 45,144 annual discharges: Hospital Clinic and Hospital de Bellvitge (greater Barcelona, Catalonia) and Hospital Juan Canalejo, La Coruña (Galicia). The two remaining hospitals: Hospital Ernest Lluch, Calatayud (Aragon), and Hospital Royo Villanova, Zaragoza (Aragon) are smaller with 122 and 238 beds and 5,800 and 8,127 annual discharges, with an urban population of 40% and 80% respectively.

Exclusion criteria were institutionalized patients, patients with nosocomial pneumonia (onset ≥2 days after hospital admission) and patients whose initial diagnosis of pneumonia was not confirmed during the hospital stay.

A case of pneumonia was defined as a patient with a chest X-ray showing pulmonary infiltrate compatible with pneumonia and one or more of the following symptoms or signs of acute infection of the lower respiratory tract: cough, pleural chest pain, dyspnea, fever >38°C, hypothermia < 35°C and abnormal auscultatory respiratory sounds unexplained by other causes.

The study was approved by the ethics committee of each participating hospital.

### Information collection and follow-up

At the initial visit, before initiation of empirical antibiotic therapy, patients underwent a complete clinical history and physical examination. A follow-up appointment was made one month after hospital discharge.

Patient information was obtained through two sources: a) Review of written hospital medical records and b) Interview of the patient or close relatives (spouse or offspring) for occupation, educational level, family situation, municipality or district of residence and smoking status using a questionnaire completed by qualified staff.

In all participating hospitals, data were collected by trained staff according to an identical protocol prepared by the working group. Clinical and sociodemographic data were collected by one person in hospital 1 and 2, by two consecutive persons at hospital 4 and 5 and by three consecutive persons at hospital 3. A pilot study was carried out to confirm the feasibility of the study protocol before the main study was initiated.

### Data measurements

The primary outcomes studied were length of stay, ICU admission, overall mortality in the first 30 days after hospital admission and readmission within 30 days after discharge. LOS was measured in days and calculated as the time from the date of hospital admission to the date of discharge.

*Socioeconomic variables *included were: individual educational level and occupation and per capita disposable family income of the municipality or district of residence (municipality family income) [municipality family income >12,500 € (high) and municipality family income ≤12,500 € (low)]. This cut-off was used because the median municipality family income in Spain in 2001 was 10,650 [17], and Aragon and Catalonia are above the national median.

The educational level was categorized as ≤primary level or ≥secondary level.

The occupation collected was the last job before retirement and was coded according to the national classification of occupations [18] - the Spanish adaptation of the British Registrar General's classification [19]: I (managers of public administrations and businesses with more than 10 employees; professions associated with post-graduate university education); II (managers of businesses with less than 10 employees; professions associated with university education; artists and sportsmen); III (civil servants, clerks and financial workers; self-employed; supervisors of manual workers); IVa (skilled manual workers); IVb (semi-skilled manual workers); and V (unskilled workers). Housewives were excluded as this information alone is not sufficiently valid to categorize the social class. The groups were further categorized as upper/middle social class (groups I-IVb) and lower class (group V). We analysed aggregations of 3 categories (I-II, III-IVb, V and I-III, IVa-IVb, V) and 2 categories (I-III versus IVa-V, I-IVa versus IVb-V) and finally chose I-IVb versus V due to the small numbers in some categories (4.1% in category I-II and 9.2% in category III) and because this best discriminated pneumonia outcomes.

The municipality family income was obtained using data from a savings bank [17] and Barcelona city council registers [20]. The median population of the municipalities or districts was 180,044 (25th percentile = 26,547, 75th percentile = 248,150). The absolute size of the municipalities ranged from 997 to 649,181.

The only city with information available by district was Barcelona, which has a total population of 1,605,602 inhabitants (INE 2006) [21]. Zaragoza, with a population of 649,181 (INE 2006) [21] had no information on the district level of municipality family income. All other municipalities had a population lower than some district of Barcelona.

#### Other variables analyzed

For each patient, information was obtained on age, sex, family situation (living alone or not), smoking status (current smoker, ex-smoker, non smoker), alcohol consumption (> 40 g/day in men, > 20 g/day in women) and the presence or absence of underlying diseases: solid or hematologic neoplasia with activity in the past year, radiotherapy in the previous three months, immunosuppressive therapy or treatment with corticosteroids ≥ 20 mg/day in the preceding month, autoimmune disease, chronic renal failure in dialysis, disabling neurological disease (neurological disease impeding daily activities), diabetes mellitus, heart failure, chronic obstructive pulmonary disease (COPD) and liver disease.

Severity of illness at presentation was quantified in five risk classes using the Pneumonia Severity Index (PSI) at admission [22]. Bacteremia, empyema, and the type of treatment (monotherapy or combined antibiotic treatment) were also collected.

### Statistical Methods

Patient characteristics and pneumonia outcomes were compared according to social status using the Chi-square test and Fisher's exact test for categorical variables and the Mann-Whitney U test for continuous variables, after assessment of non-normal distribution.

Multivariate logistic regression analysis was used to investigate the relationship between socioeconomic status and outcome variables. Variables with a p < 0.2 in the bivariate analysis, sex, and socioeconomic variables including education, were introduced in the multivariate model, whenever they were not redundant; municipality family income and social class were introduced separately in the model. In the final adjusted model, only variables with a p < 0.05 were included. Odds ratios (OR) and their corresponding 95% confidence intervals (CI) were calculated.

All statistical tests were two-tailed and statistical significance was established as p < 0.05.

The model including LOS as the dependent variable was adjusted with a generalized linear model (GLM) using negative binomial regression which is appropriate for modeling overdispersed data [23]. Estimated coefficients (ec) were calculated to observe the relationship between variables; an ANOVA test was used to select the best model. In the adjusted model, only variables with p < 0.05 or non-significant variables that showed a significance of second order iteration were retained. The statistical analysis was performed using the SPSS v.15.0 and R v.2.10.1 statistical programs.

## Results

### Characteristics of the Study Population

Of the 695 patients with pneumonia recruited, the municipality family income could not be obtained in 44 patients, who were excluded. The characteristics of the 651 remaining patients and their distribution by admitting hospital are shown in table 1. The median age was 77 years (range: 65-100) and 62.1% were male. One hundred and fifteen patients (17.7%) lived in a municipality or district with a high municipality family income. Only 101 of patients (15.7%) had a secondary education. A total of 396 (60.8%) patients were admitted to hospitals 2 and 3. A total of 7.8% of patients were admitted to the ICU, 6.3% died in the first 30 days after admission and 10.6% were readmitted within 30 days after discharge. The median hospital stay was 8 days.

**Table 1:** Characteristics and outcomes of the study population.

		Patients studied n	n	(%)
Sex: male		651	404	(62.1)
Age (years)	M (range)	651	77 (65-100)	
High MFI		651	115	(17.7)
Social class		511		
	I		14	(2.7)
	II		7	(1.4)
	III		47	(9.2)
	IVa		183	(35.8)
	IVb		74	(14.5)
	V		186	(36.4)
Educational level	≥secondary level	643	101	(15.7)
Living alone		651	77	(11.8)
Alcohol consumption		561	51	(9.1)
Smoker or ex-smoker		648	342	(52.8)
Admitting hospital		651		
	1		117	(18.0)
	2		163	(25.0)
	3		233	(35.8)
	4		48	(7.4)
	5		90	(13.8)
Underlying diseases		651	460	(70.7)
*COPD*			221	(33.9)
*Diabetes mellitus*			130	(20.0)
*Solid or haematologic neoplasia*			96	(14.8)
*Disabling neurological disease*			86	(13.2)
*Heart failure*			64	(9.8)
*Aggressive therapy*^a^			48	(7.4)
*Liver disease*			21	(3.2)
*Other diseases*^b^			29	(4.4)
Pneumonia Severity Index		589		
	Risk class 1		2	(0.3)
	Risk class 2		39	(6.6)
	Risk class 3		173	(29.4)
	Risk class 4		286	(48.6)
	Risk class 5		89	(15.1)
Combined antibiotic treatment		651	251	(38.6)
Bacteremic pneumonia		405	55	(13.6)
Empyema		651	17	(2.6)
**Pneumonia outcomes**				
LOS (days):	M (range)	651	8 (1-95)	
Mortality in the first 30 days		651	41	(6.3)
Readmission ^c^		604	64	(10.6)
ICU admission		651	51	(7.8)

Data are presented as n (%) or median (range).

Abbreviations: COPD: Chronic obstructive pulmonary disease; ICU: Intensive Care Unit; LOS: length of stay; M: Median; MFI: Municipality Family Income.

Notes: ^a ^Aggressive therapy: radiotherapy or corticosteroids therapy or immunosuppressive therapy.

^b ^Other diseases: Autoimmune disease or chronic renal failure in dialysis.

^c ^In order to evaluate readmission, deaths during hospitalization were excluded.

The occupation was categorized in 511 patients: 63.6% were class I-IVb and 36.4% group V. All patients answered the question about last stated occupation and none said they were homeless.

Information about social class was not obtained in 140 (21.5%) patients, of whom 91.4% were women and 86.4% were housewives, with a mean age of 77 (65-96) years, which did not differ from that of patients on whom information was available (p = 0.724). In municipalities with a high municipality family income, 20% of inhabitants had low social class versus 40% in municipalities with a low municipal family income (p < 0.001).

### Statistical Analysis

Table 2 compares patient characteristics, distribution by admitting hospital and pneumonia outcomes according to municipality family income, social class and educational level. There were significant differences in patient distribution by admitting hospital according to municipality family income, social class and educational level (p < 0.001).

**Table 2:** Patient characteristics, admitting hospital and outcomes of pneumonia according to municipality family income, educational level and social class.

		Municipality Family Income					Educational level					Social Class				
		(n = 651)					(n = 643)					(n = 511)				
		**High MFI**		**Low MFI**			**≤ primary**		**≥secondary**			**Class I-Vb**		**Class V**		
		**n = 115**		**n = 536**			**n = 542**		**n = 101**			**n = 325**		**n = 186**		
		**n**	**(%)**	**n**	**(%)**	**p**	**n**	**(**%**)**	**n**	**(%)**	**p**	**n**	**(%)**	**n**	**(%)**	**p**

Sex: male		69	(60.0)	335	(62.5)	0.616	317	(58.5)	82	(81.2)	≤0.001	269	(82.8)	123	(66.1)	≤0.001
Age (years)	M (range)	78 (66-96)		76.5 (65-100)		0.075	77 (65-100)		76 (65-94)		0.033	76 (65-96)		78 (65-100)		0.029
Living alone		22	(19.1)	55	(10.3)	0.008	65	(12.0)	12	(11.9)	0.975	37	(11.4)	21	(11.3)	0.974
Alcohol consumption		3	(2.6)	48	(10.7)	0.007	44	(9.5)	7	(7.2)	0.470	35	(12.3)	14	(8.7)	0.245
Smoker or ex-smoker		64	(55.7)	278	(52.2)	0.496	260	(48.1)	77	(76.2)	≤0.001	228	(70.4)	91	(48.9)	≤0.001
Admitting Hospital						≤0.001					≤0.001					≤0.001
	1	102	(88.7)	15	(2.8)		75	(13.8)	42	(41.6)		78	(24.0)	18	(9.7)	
	2	4	(3.5)	159	(29.7)		150	(27.7)	13	(12.9)		104	(32.0)	36	(19.4)	
	3	0	(0)	233	(43.5)		189	(34.9)	36	(35.6)		91	(28.0)	90	(48.4)	
	4	0	(0)	48	(9.0)		44	(8.1)	4	(4.0)		21	(6.5)	16	(8.6)	
	5	9	(7.8)	81	(15.1)		84	(15.5)	6	(5.9)		31	(9.5)	26	(14.0)	
Underlying diseases^a^		77	(67.0)	383	(71.5)	0.336	385	(71.0)	67	(66.3)	0.343	238	(73.2)	128	(68.8)	0.287
*Heart failure*		18	(15.7)	46	(8.6)	0.021	54	(10.0)	10	(9.9)	0.985	33	(10.2)	14	(7.5)	0.323
*COPD*		26	(22.6)	195	(36.4)	0.005	189	(34.9)	29	(28.7)	0.230	134	(41.2)	64	(34.4)	0.128
PSI	RC ≥ 4	54	(73.0)	321	(62.3)	0.075	312	(62.5)	55	(67.1)	0.429	193	(67.2)	103	(59.9)	0.111
Combined antibiotic treatment		58	(50.4)	193	(36.0)	0.004	209	(38.6)	39	(38.6)	0.992	132	(40.6)	65	(34.9)	0.205
Bacteremic pneumonia^b^		12	(14.5)	43	(13.4)	0.794	43	(13.0)	11	(15.7)	0.551	30	(13.7)	12	(11.0)	0.492
Empyema		5	(4.3)	12	(2.2)	0.200	13	(2.4)	4	(4.0)	0.323	10	(3.1)	4	(2.2)	0.537
**Pneumonia outcomes**																
LOS (days)	M (range)	8 (3-51)		8 (1-95)		0.464	8 (1-93)		7 (2-95)		0.387	8 (1-95)		8 (2-57)		0.306
Mortality		8	(7.0)	33	(6.2)	0.749	32	(5.9)	5	(5.0)	0.706	22	(6.8)	5	(2.7)	0.047
Readmission ^c^		14	(13.3)	50	(10.0)	0.316	49	(9.7)	15	(15.6)	0.086	34	(11.4)	20	(11.0)	0.827
ICU		19	(16.5)	32	(6.0)	≤0.001	39	(7.2)	9	(8.9)	0.547	28	(8.6)	7	(3.8)	0.037

Data are presented as n (%) or median (range).

Abbreviations: COPD: Chronic Obstructive Pulmonary Disease; ICU: Intensive Care Unit; LOS: Length of stay; M: Median; MFI: Municipality Family Income; PSI: Pneumonia Severity Index.; RC: Risk Class.

Notes: ^a ^Only underlying diseases with different distribution between two groups of municipality family income, educational level or social class are listed.

^b ^Only 405 patients in whom blood cultures were made were evaluated.

^c ^In order to evaluate readmission, deaths during hospitalization were excluded.

Length of stay and readmission were identical according to social class, educational level and municipality family income (table 2). However, ICU admission was greater in patients with a high municipality family income (16.5%) than in those with a low municipality family income (6.0%) (p < 0.001) and in those in social class I-IVb (8.6%) compared with class V (3.8%) (p = 0.037). Mortality was also higher in patients in social class I-IVb (6.8%) compared with social class V (2.7%) (p = 0.047).

Table 3 and 4 show the results of the crude and the adjusted analysis according to ICU admission and mortality respectively. Adjusted analysis showed no association between these outcomes and social class or municipality family income. We found no association between outcomes and educational level.

**Table 3:** Crude and adjusted analysis according to ICU admission.

	Group	N ICU/N group	Crude OR (95% CI)	P value	Adjusted OR (95% CI)	P value
Age (years): Median (range)	ICU no	77 (65-100)				
	ICU yes	75 (65-87)	--	0.007	0.91 (0.86-0.96)	0.001
Municipality Family Income	Low	32/536				
	High	19/115	3.12 (1.70-5.71)	<0.001	--	--
Social Class	V	7/186				
	I-IVb	28/325	2.41 (1.04-5.65)	0.037	--	--
Disabling Neurological Disease	no	50/565				
	yes	1/86	0.12 (0.02-0.89)	0.013	**--**	--
PSI	<4	4/214				
	≥4	42/375	6.62 (2.34-18.74)	<0.001	5.80 (2.01-16.69)	0.001
Combined antibiotic Treatment treatment	no	12/400				
	yes	39/251	5.95 (3.05-11.60)	<0.001	6.37 (2.89-14.05)	<0.001
Radiotherapy	no	49/647				
	yes	2/4	12.20 (1.68-88.52)	0.033	--	--
Bacteremia^a^	no	32/350				
	yes	11/55	2.48 (1.17-5.28)	0.015	--	--
Empyema	no	47/634				
	yes	4/17	3.84 (1.20-12.25)	0.037	--	--
Admitting Hospital						
	1	23/117	Ref		Ref	
	2	14/163	0.38 (0.19-0.78)	0.011	0.21 (0.09-0.51)	0.001
	3	10/233	0.18 (0.08-0.40)	<0.001	0.15 (0.06-0.39)	<0.001
	4	4/48	0.37 (0.12-1.14)	0.104	0.74 (0.24-2.28)	--
	5	0/90	0.02 (0.00-0.37)	<0.001	NC	--
LOS (days): Median (range)	ICU no	8 (1-95)				
	ICU yes	15 (1-62)	--	<0.001	1.05 (1.03-1.08)	<0.001

Abbreviations: CI: Confidence interval; ICU: Intensive Care Unit; LOS: Length of stay; M: Median; NC: Not Calculated; OR: Odds Ratio;

PSI: Pneumonia Severity Index.

^a ^Only 405 patients in whom blood cultures were made were evaluated.

For ICU admission we adjusted by age, PSI, combined antibiotic treatment, hospital admission and length of hospitalization.

**Table 4:** Crude and adjusted analysis according to mortality.

	Group	N mortality/N group	Crude OR (95% CI)	P value	Adjusted OR (95% CI)	P value
Age (years): Median (range)	mortality no	77 (65-100)				
	mortality yes	80 (65-96)	--	0.017	1.07 (1.00-1.14)	0.047
Social Class	V	5/186				
	I-IVb	22/325	2.63 (0.98-7.04)	0.047	--	--
PSI	<4	4/214				
	≥4	34/375	5.24 (1.83-14.96)	0.001	--	--
Underlying diseases	no	3/191				
	yes	38/460	5.64 (1.72-18.51)	0.001	--	--
Disabling Neurological Disease	no	29/565				
	yes	12/86	3.00 (1.47-6.13)	0.002	5.00 (1.58-15.83)	0.006
Haematologic neoplasia	no	32/607				
	yes	9/44	4.62 (2.05-10.43)	0.001	7.20 (2.29-22.66)	0.001
Chronic renal failure	no	39/639				
	yes	2/12	3.08 (0.65-14.53)	0.171	6.59 (1.18-36.94)	0.032
Radiotherapy	no	39/647				
	yes	2/4	15.59 (2.14-113.65)	0.021	--	--
Liver disease	no	37/630				
	yes	4/21	3.77 (1.21-11.78)	0.037	--	--
Bacteremia^a^	no	20/350				
	yes	10/55	3.67 (1.61-8.33)	0.003	2.82 (1.11-7.15)	0.029
ICU	no	30/600				
	yes	11/51	5.22 (2.44-11.19)	<0.001	13.38 (4.64-38.59)	<0.001

Abbreviations: CI: Confidence interval; ICU: Intensive Care Unit; OR: Odds Ratio; PSI: Pneumonia Severity Index.

^a^Only 405 patients in whom blood cultures were made were evaluated.

For mortality we adjusted by age, disabling neurological disease, haematologic neoplasia, chronic renal failure, bacteremia and ICU.

Adjusted analysis with readmission as the dependent variable showed no association between readmission and any socioeconomic factor. Suffering an underlying disease was the only factor associated with readmission [OR: 4.79 (2.03-11.32); p < 0.001].

Negative binomial regression showed that LOS did not change according to socioeconomic level or social class. LOS increased according to ICU admission (ec:0.698; p < 0.001), PSI ≥ 4 (ec:0.164; p = 0.004), and empyema (ec:0.455; p = 0.004). Being a smoker or ex-smoker non-significantly increased LOS (ec:0.007; p = 0.895), whereas living alone non-significantly reduced it (ec:-0.221; p = 0.062). However, LOS increased significantly in patients in whom both factors coincided (ec:0.608; p < 0.001).

## Discussion

This study found no association between social class or socioeconomic status and pneumonia outcomes.

A French study by Stelianides *et al. *[10] in patients hospitalized with CAP found no relationship between low socioeconomic status and greater disease severity; ICU admission and deaths attributed to pneumonia were identical in groups with high and low socioeconomic status. However low socioeconomic status was associated with a longer hospital stay not explained by clinical factors and the authors suggested that underprivileged social status leads to extended hospital stays to ensure compliance with treatment and a favorable evolution of pneumonia. Our study population included patients aged ≥65 years, compared with patients aged >18 years in the study by Stelianides *et al. *[10], which might explain the differing results found. Furthermore, in the French study, socioeconomic status was classified differently: low socioeconomic status included the long-term unemployed, the homeless and persons living in unhealthy or overcrowded conditions and those totally-dependent on government welfare; if none of these conditions was present, socioeconomic status was classified as average. In contrast, we classified socioeconomic status according to patient occupation and although 36.4% of our patients were in social class V, they did not possess these marginal characteristics, as shown by the answers to questions on occupation and homelessness.

A Canadian retrospective cohort study in the elderly by Vrbova *et al.*[12] concluded that lower socioeconomic status did not increase CAP mortality. The definition of socioeconomic groups was closer to ours, although they only evaluated socioeconomic status using the municipality family income and not by individual social class. Another Canadian study by McGregor *et al.*[11] examined the relationship between socioeconomic status and length of hospitalization and hospital readmission and found that people suffering economic hardship (pensioners) had a greater risk of earlier readmission and a non-significant longer median hospital stay. However, social class was categorized differently, including a high percentage of socially marginalized patients (34%) in the lowest social class, which could have explained the differences with our results. When they characterized socioeconomic status according to neighborhood income, no association was found, as in our study.

Jasti *et al. *[13] studied risk factors for readmission of patients hospitalized with CAP and found that less than a high school education, unemployment, coronary artery disease and COPD were independently associated with rehospitalisation. We found no association between rehospitalisation and low educational levels, COPD, social class or municipality family income.

Like Stelianides *et al*. [10], we found no association between social class and ICU admission. We observed an association in the crude analysis between ICU admission and upper/middle social class or high municipality family income, which may be due to the higher proportion of patients with high socioeconomic status coming from one hospital, which had most ICU admissions (p < 0.001). In the multivariate analysis adjusted by admitting hospital, the association between ICU and social class or municipality family income disappeared, but the association between ICU and admitting hospital persisted. The multivariate analysis also showed an association between ICU admission and a longer hospital stay and higher mortality, in line with other reports [24,25]. The negative association between ICU admission and older age might be explained by a less interventionist attitude towards some types of patients.

The mortality rate in CAP requiring hospitalization was 6.3%, lower than the reported by Monge *et al*, Marston *et al. *and Zalacain *et al. *in patients aged ≥65 years (12%, 12.5% and 11% respectively) [6,7,26]. However Venditti *et al. *[27] reported a similar mortality in patients hospitalized with CAP (6.7% in patients with a median age of 73.9 years). We report similar figures to a European study, but slightly lower than other Spanish and American studies.

Several factors support the robustness of our methods. Patient occupation was obtained directly from the patient or close relative, in contrast to other studies which used administrative databases [11,12]. In addition, compared to other studies on hospitalized patients with CAP [10], we evaluated the socioeconomic status in a larger number of patients. Lastly, patients were recruited from five public hospitals providing universal free health care to the whole population, which guaranteed that all social classes were represented.

The limitations of our study include a possible bias due to the exclusion of patients whose economic level or social class could not be coded. Housewives were also excluded as this information alone was not considered sufficiently valid to categorize social class according to occupation; it might have been useful to categorize the social class of heads of households for housewives not living alone, but this was not done. However, patient characteristics and CAP outcomes were compared in study subjects and those excluded and no significant differences were found. Pneumonia outcomes were analyzed in women excluded from the study (mortality: 7.8%, ICU:7.8%; LOS: 8 (1-85); readmission: 7.7%) and compared with women included (mortality:5.0%, ICU:5.9%; LOS:8 (1-57); readmission: 8.8%) with no significant differences being found (mortality: p = 0.377, ICU: p = 0.549; LOS: p = 0.871; readmission: p = 0.750). Before excluding housewives from the sample, there was a higher percentage of men (62.1) than women, confirming other reports [10,25,28]. Another possible limitation is that most patients were retired and it is difficult to measure social class in the elderly; however the last occupation of retired people is also accepted as a means of categorizing social class [19]. Likewise, the municipality family income was collected as a group variable and this has also been accepted as a means of categorizing economic status in other studies [11,12]. Another possible limitation is that municipality family income by district was only available in Barcelona city, unlike other studies [29], with only municipal data available for the remaining patients. However, only one municipality (Zaragoza) had a higher population than the most-populous district of Barcelona and only accounted for 64 patients (9.8% of the total). This may explain why no association was found. Future research may need to consider infra-municipality variables.

## Conclusions

We measured socioeconomic status using both individual and community data and found no association between social class, educational level or municipality family income and the variables of pneumonia outcomes. The lack of differences in pneumonia outcomes between social classes supports the provision of universal, equitable health care by the public health system. The length of stay significantly increased in patients who lived alone and were smokers or ex-smokers.

Seven point eight per cent of patients hospitalized for CAP were admitted to the ICU, 6.3% died in the first 30 days after admission and 10.6% were readmitted within 30 days after discharge. The median hospital stay was 8 days.

## Competing interests

The authors declare that they have no competing interests.

## Authors' contributions

CI contributed to the conception and design of the study, interpretation of data and drafting of the manuscript. MO contributed to the statistical analysis. LR contributed to the preparation of the data base and statistical analysis. XS contributed to the investigation of socioeconomic variables of patients and preparation of the data base. IV contributed to the codification of patient occupations. MN, JMB, JC, WV, DS, JMC, and LS contributed to the study design and validation of data. AD contributed to the conception and design of the study, interpretation of the data, and coordination of the study. All authors have revised and approved the final version of the manuscript.

## Pre-publication history

The pre-publication history for this paper can be accessed here:

http://www.biomedcentral.com/1471-2458/10/421/prepub
